# Proactive approach for preamble detection in 5G-NR PRACH using supervised machine learning and ensemble model

**DOI:** 10.1038/s41598-022-12349-4

**Published:** 2022-05-19

**Authors:** Syeda Sundus Zehra, Maurizio Magarini, Rehan Qureshi, Syed Muhammad Nabeel Mustafa, Faiza Farooq

**Affiliations:** 1 Sir Syed University of Engineering and Technology, Karachi, Pakistan; 2 Politecnico di Milano, Milan, Italy; 3 NED University of Engineering and Technology, Karachi, Pakistan

**Keywords:** Engineering, Mathematics and computing

## Abstract

The physical random access channel (PRACH) is used in the uplink of cellular systems for initial access requests from the users. It is very hard to achieve low latency by implementing conventional methods in 5G. The performance of the system degrades when multiple users try to access the PRACH receiver with the same preamble signature, resulting in a collision of request signals and dual peak occurrence. In this paper, we used two machine learning classification technique models with signals samples as big data to obtain the best proactive approach. First, we implemented three supervised learning algorithms, Decision Tree Classification (DTC), naïve bayes (NB), and K-nearest neighbor (KNN) to classify the outcome based on two classes, labeled as ‘peak’ and ‘false peak’. For the second approach, we constructed a Bagged Tree Ensembler, using multiple learners which contributes to the reduction of the variance of DTC and comparing their asymptotes. The comparison shows that Ensembler method proves to be a better proactive approach for the stated problem.

## Introduction

During the last decade, there have been an evolution is the wireless technology. The rapid advancements in this technology have massively changed the loves of the people. Since the inception of First generation (1G), cellular networks, generations have been launched with enormous difference in terms of network architecture, mobility, privacy and security. Both academic and corporate communities are already working feverishly to complete 5G standardization and commercialization by the end of 2019^1^. 5G is planned to be a key enabler and a leading infrastructure provider in the information and communication technology industry by enabling a variety of upcoming services with different requirements, driven by the desire to meet today’s expanding mobile traffic. Fifth Generation (5G) cellular networks provides key enabling technologies for the ubiquitous deployment of modern technologies. These includes enhanced mobile broadband (eMBB) communication, massive Machine type communication (mMTC) and ultra-reliable low-latency communication (URLLC)^2^.

The spectrum of a signal in 4G-LTE and 5G-NR systems changes from time to time in symbol periods in units of resource blocks (RBs) in the frequency domain^3^. 5G is enhanced version of 4G Long Term Evolution (LTE), New Radio (5G-NR) with features like numerology or sub-carrier spacing Orthogonal Frequency Division Multiple Access (OFDMA), massive Multiple Input Multiple Output (MIMO), beamforming and Millimeter Wave (mmWave) are introduced in 5G to obtain high speed, improved and more reliable performance than LTE. To meet the increasing number of wireless devices and demand for high data rates, the MIMO (multiple inputs-multiple outputs) technology is widely used to provide a solution for the next decade^4^. MIMO is a technology based on multiple antennas which is mainly used for wireless communication. Multiple antennas are combined at both ends; transmitter and receiver which aids to minimize errors and improve data speed. 5G MIMO is used in multiple scenarios like heterogeneous networks, automotive networks, and millimeter-Wave networks^5^. In terms of user density, latency, and speed, 5G is predicted to greatly beat current LTE^6^. Radio access in 5G-NR is even more complicated and involved than in 4G long term evolution (LTE) and LTE-Advanced. As a result, the 5G-NR requirements are fairly detailed^7^ .

Specifications are brief, design objectives are rarely addressed, and information is often muddled or dispersed over multiple pages. Dealing with these needs control over physical layer of the network^8^. As far as PRACH procedure for 5G is concerned, it is the same as that of 4G specifically when targeting on the issue of initial access a solution that first comes into mind is to implement the same technique used in LTE on 5G system but with slight changes to detect the presence of a User Equipment (UE)^9,10^. PRACH is a technique through which a User Equipment can establish a connection with the gNB, or base station. Each base station in today’s LTE networks contains up to 64 orthogonal ZC sequences, and a UE selects one at random to transmit over the PRACH channel. All the techniques assure the detection of 99% correct preamble with signal-to-noise ratio (SNR) values greater than − 14.2 dB, according to 3GPP standard^11^. As 5G requires a high level of accuracy and speed to rule over the world, in order to make this possible the system must adapt mechanism to use latest problem handling techniques, that could be achieve with the blending of Artificial Intelligence(AI) in 5G^10^.

AI procedures are required in order for a 5G network to be fully operational and efficient. Existing 4G networks with all-IP (Internet Protocol) broadband access are built on a reactive model, which results in low spectrum efficiency. However, many general as well as complicated cases in communication systems can be seen in the literature providing solutions to issues that have already been addressed in contrast with different AI approaches^10^. In these days ML can be seen as a new trend in the tech world. Several PRACH related issues have already been addressed by using ML^12^. So, in order to achieve better performance we considered and looked into many solutions and came up with ML based approach for efficient preamble detection in PRACH.

**Figure 1:**
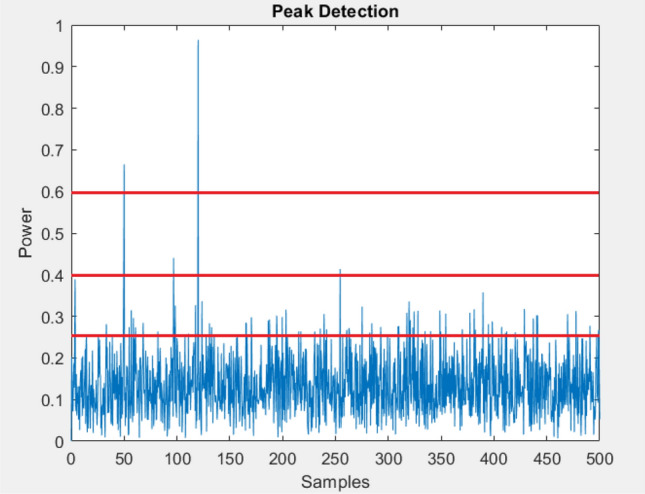
Peak detection.

As a subject, ML has progressed to the point that it now allows wireless networks to learn and extract knowledge by interacting with data. Engineers and researchers all around the world have expressed preliminary interest in and conversations regarding the potential of evolving 5G standards with the help of machine learning protocols^10^. For uplink initial access/synchronization, PRACH supports several long and short formats for different coverage and combinations of formats with configurable time/frequency placements, as shown in Fig. 1. A PRACH preamble can have varying number of sequence repetitions depending on the format to provide different coverage range. Preambles are sent in PRACH slots with customizable periods in the time domain^13^. A PRACH slot can contain a set of PRACH occasions in frequency domain, depending on system setup each PRACH occasion has a preamble sequence. In ML any fix and specific rule is not defined to follow from beginning till the end. We start by checking the data values and class labels and then choosing the branch of ML according to the nature of collected data. Data values can be categorized mainly into two types i.e. continues values or discrete values^14^. In our particular scenario the collected class labels are discrete in nature and can be classified as a binary class problem, making us to choose supervised learning and in addition we tried implementing ensemble method in order to achieve more accuracy and making our system more robust. Initially we performed training and testing on many different classification models and came up with three techniques that were more accurately classifying false peak and the true peak as compare to the other techniques. The selected techniques were DTC, KNN and NB.

In this paper, the organization is as follows, “Background” section discusses the background of 5G PRACH problem. Conventional way of solving this problem and solving the same problem by applying Supervised ML and Ensemble Method is discussed in “Conventional preamble detection method” section. Whereas in “Experimental setup and results” section the experimental setup and execution results of implemented algorithms are discussed. Finally the work is concluded in “Conclusion” section.

## Background

Telecommunication industry is rapidly growing and 5G these days is widely integrated with AI to achieve its key enablers^10^. AI has many approaches like Natural Language Processing (NLP), ML, Computer Vision and many more being used in wireless communication, learning and adapting human behaviors, enabling self-organization in networks are different perspectives covered in AI-defined 5G^12^. AI-defined 5G network handles real-time data at Base Stations^14^. ML is one of the approaches of AI. ML algorithm learns information from data by using probabilistic and statistical approaches and used to classify big data into subclasses or labels, predict results based on learned patterns. Recurrent Neural Network (RNN), Deep Belief Network (DBN) and Convolution Neural Network (CNN) are different types of Neural Networks used in telecommunication problem^15,16^. These are data driven approaches and have been used to perform non-linear approximations for optimization in wireless communication systems^14^. ML has been already used in PRACH to improve the performance in collision detection and to reduce missed detection probability, load and latency. In 5G initial access/PRACH preamble of a UE to a base station requires uplink synchronization between the sender and the receiver to obtain orthogonality, a PRACH preamble signature with a specific pattern is assigned to a UE, after being synchronized with each other message communication can be done. The uplink random access method, which includes TA, is the subject of Ref.^17^. The random access channel (RACH) processes for 5G NR networks are explored in particular, as well as the impact of their deployment on satellite links.

Authors in Ref.^18^ have presented a model that uses supervised machine learning in preamble detection neural networks are presented as a way for modeling the PRACH signal in the base station receiver in this paper which is a problem of supervised learning regression. A neural network is trained to learn the relationship between the inputs, which are selected parameters that describe the situation, and the output, which is an I/Q signal. In Ref.^19^, authors have presented an ML-based model for predicting successful preamble broadcasts at a base station and as a result, forecasting the appearance of congestion under bursty traffic situations in this work. A RNN ML technique is used in the model, which is based on the long short-term memory architecture. This article^7^ lays out all of the design specifics for initial access channels and signal generation in 5G-NR standards. The paper’s contributions are threefold, design specifics and rationale for both downlink and uplink access channels, as well as signal generation information are described first. Secondly, receiver design aspects of NR PRACH short formats are discussed. Lastly, PRACH receiver implementation aspects and performance reports from different network operators are presented and compared with 3GPP specified Radio Performance and Protocol aspect requirements for millimeter wave (mmW) access. Table 1 shows the existing state-of-art work.

**Table 1:** Implementation of Machine learning in the domain of telecommunication.

Articles	Telecommunication area	Machine learning approaches
Classification of indoor environments for IoT applications: a machine learning approach^20^	Real-time measurement for the RF signal	Machine learning
Network traffic prediction based on diffusion convolutional recurrent neural networks^15^	To forecast traffic load on the links of a real backbone network	Diffusion convolutional recurrent neural network (DCRNN)
Network traffic prediction based on deep belief network in wireless mesh backbone networks^16^	Proposed a network traffic prediction method based on a deep belief network	Deep belief network
Modelling PRACH signals in base station with neural network	To predict the PRACH data signal	Neural network
Supervised learning based arrival prediction and dynamic preamble allocation for bursty traffic^21^	To predict dynamic preamble allocation for bursty traffic	Supervised machine learning
Preamble transmission prediction for mMTC bursty traffic: a machine learning based approach^19^	Predicting successful preamble transmissions at a base station and subsequently forecasting the possible occurrence of congestion under bursty traffic conditions	Machine learning
A machine learning-based design of PRACH receiver in 5G^11^	To predict PRACH Preamble detection	Supervised machine learing

**Table 2:** Comparison with the existing state-of-art work.

Article	Year	Problem	Solution
A PRACH preamble generation and detection model for 5GNR systems^22^	2021	Preamble detection probability, miss-detection probability, false alarm probability and null	Provides the orthogonal frequency division multiple access (OFDM) baseband signal generation and detection method for physical random-access channel (PRACH)
Preamble design and collision resolution in a massive access IoT system^23^	2021	Support massive access efficiently is one of the challenges in the future Internet of Things (IoT) systems	An effective preamble collision resolution scheme to sustain massive random access (RA) for an IoT system
A proposed preamble detection algorithm for 5G-PRACH^13^	2019	Detection of preambles in physical random access channel (PRACH) between user equipment (UE) and next generation Node B (gNB)	Method for detecting preamble in PRACH at 5G base station (gNB)
Modelling PRACH signals in base station with neural network^18^	2020	Detection of preamble in PRACH	Neural networks as a method for modelling the PRACH signal in the base station receiver
Enabling LTE PRACH collision multiplicity detection via machine learning^24^	2019	Base Stations (eNBs) cannot infer the number of collided User equipments (UEs)	Machine learning techniques to design a system that is able to estimate the collision multiplicity and thus gather information about devices chose the same preamble
A machine learning-based design of PRACH receiver in 5G^11^	2019	Preamble detection	Performance degradation in the extension of the 4G technique to 5G

But there are cases when preamble request could be made from two or more UEs at the same time and these conflicting UEs could randomly select an identical signature, this problem occurs at lower SNR values, due to which a receiver might be confused for the presence of a user. To solve this issue, the network itself will detect that which preamble signature to be used and when. Here the point of concern is that how a network would differentiate between the preambles? The comparison of our work with the existing state-of-art work is elaborated in Table 2.

## Conventional preamble detection method

**Figure 2:**
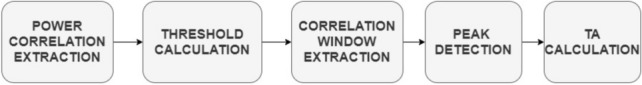
Classic detection algorithm.

Preambles are generated using Zadoff-Chu (ZC) sequence in 5G^11^. The conventional technique used for preamble detection uses a window-based detection approach due to correlation properties of ZC^25^. The presence of a preamble is verified for each window by comparing every sample with the detection threshold, that enables PRACH preamble detection in an efficient and adequate manner, based on managing power delay profile using periodic correlation. Reduction and optimization of intra-cell interference between different preambles is done in order to achieve better detection. The classical method consists in extracting power correlation, Calculating threshold, extracting correlation window, finding the highest value that exceeds a pre-calculated threshold for every searching window, i.e. the peak value detection and the position of the highest value in the search window that represents the delay of the preamble^26^. The basic idea of the conventional detection algorithm is shown in Fig. 2.

In the above method there was a problem of missed detection and because of this we face more false peaks.

### Proposed supervised learning-based approach for preamble detection

Implementation of conventional method for preamble detection used in 4G LTE and LTE-A on 5G resulted in false peak generation at the receiver end, which was the main reason of performance degradation in 5G system. The concept of using ML techniques to improve performance in PRACH for preamble detection is not new and we are well aware with the capability of ML which grants to use smart algorithms for classification and prediction^11^. Here we choose supervised learning method that classifies false and true peaks and enables elimination of false peaks.

We have implemented three supervised machine learning (SML) algorithms. In order to choose a best out of these three for preamble detection in PRACH by comparing prediction accuracy of these algorithms. DTC with 4 splits is used as it is one of the most commonly used SML algorithm for binary classification and it completely fits in our use case scenario of classifying two classes namely ‘Peak’ and ‘FalsePeak’.

We have implemented three SML algorithms. In order to choose a best out of these three for preamble detection in PRACH by comparing prediction accuracy of these algorithms. DTC with 4 splits is used as it is one of the most commonly used SML algorithm for binary classification and it completely fits in our use case scenario of classifying two classes namely ‘Peak’ and ‘FalsePeak’.

Steps to perform DTC^27^:

1. First step is to calculate entropy for both the classes.
  $$\begin{aligned} Entropy(Class)= & {} -P/N+P[\log _{2}(P/N+P)]\\&-N/N+P[\log _{2}(N/N+P)]. \end{aligned}$$
2. Then to calculate information gain for each feature:
  $$\begin{aligned} I(P_{i},N_{i})= & {} -P/N+P[\log _{2}(P/N+P)]\\&-N/N+P[\log _{2}(N/N+P)]. \end{aligned}$$
3. Determine entropy of each individual feature:
  $$\begin{aligned} Entropy(x) = \sum (P_{i} + N_{i}) \times [I(P_{i},N_{i})]. \end{aligned}$$
4. Final step is to calculate final gain:
  $$\begin{aligned} Gain = Entropy(class)-Entropy(x). \end{aligned}$$

The second technique we used is NB, that uses Bayesian theorem to calculate probabilities of classes by adjusting with different distribution methods. We used Gaussian distribution as per need of our case.

To perform NB it is important to calculate posterior probability:

$$\begin{aligned} P(h|D) = P(D|h)p(h) / p(D), \end{aligned}$$

where, P(h) $=$ Probability of hypothesis, P(D) $=$ Probability of data, P(h|D) $=$ Probability of hypothesis ‘h’ given data ‘D’ & P(D|h) $=$ Probability of data ‘D’ when hypothesis ‘h’ is true^28^.

Lastly we used KNN technique on our discrete type of dataset and kept the value of $\hbox {k}=10$ neighbors, calculating the distance of K by each data value using Euclidean method.

$$\begin{aligned} d=\sqrt{(x_{1}-x_{2})^{2} + (y_{1}-y_{2})^{2}}. \end{aligned}$$

**Figure 3:**
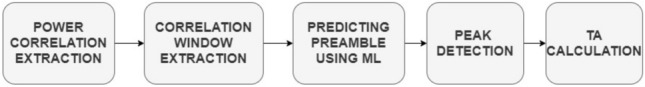
Machine learning preamble detection.

The flow can be seen in Fig. 3, which depicts proposed method of using machine learning for detection.

Furthermore, we proposed a second model which is based on Ensemble methods for preamble detection. It also helps in reducing variance and avoids over-fitting.

### Implementing bagged tree ensemble method

**Figure 4:**
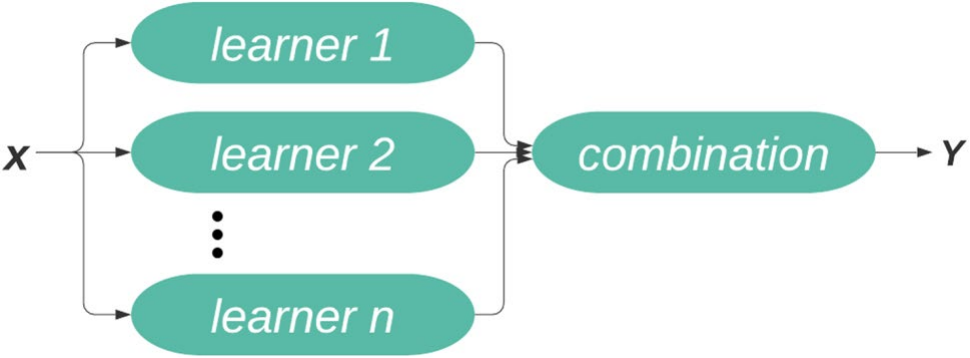
Architecture of ensemble.

Ensemble methods are regarded as state-of-art solutions for the numerous machine learning challenges^29,30^. Ensembles are groups of learning machines that combine their decisions, learning algorithms, data views, and other basic characteristics to make more consistent and accurate predictions in supervised and unsupervised learning problems. Ensemble methods construct a collection of classifiers and then use weighted vote of their predictions to classify new data points^31^. In Fig. 4, common ensemble architecture is shown. The base of an ensemble constitutes of a number of learners. Base learners are usually generated by base learning algorithms which could be any ML algorithm. This method assists in enhancing the performance of single model by training multiple models and then give combined predictions. Ensemble methods improve the quality of predictions by minimizing the variance of estimators^32^.

The most effective technique to create an ensemble is the Bagging algorithm. Bagging is a simple and intuitive approach that produces good results while reducing variance and avoiding over-fitting^33^. The ensemble created by this algorithm combines learners into a single classifier. Bagging can be done in parallel to keep a check on excessive computational resources. In bagging, bootstraps are first created either by regression or classification algorithm. Bagging is represented by the following formula:

$$\begin{aligned} f_{bag} = f_{1}(x) + f_{2}(x) + \cdots + f_{30}(x)). \end{aligned}$$

The term on the left-hand side is the bagged prediction and terms on the right-hand side are the individual learners.

**Figure 5:**
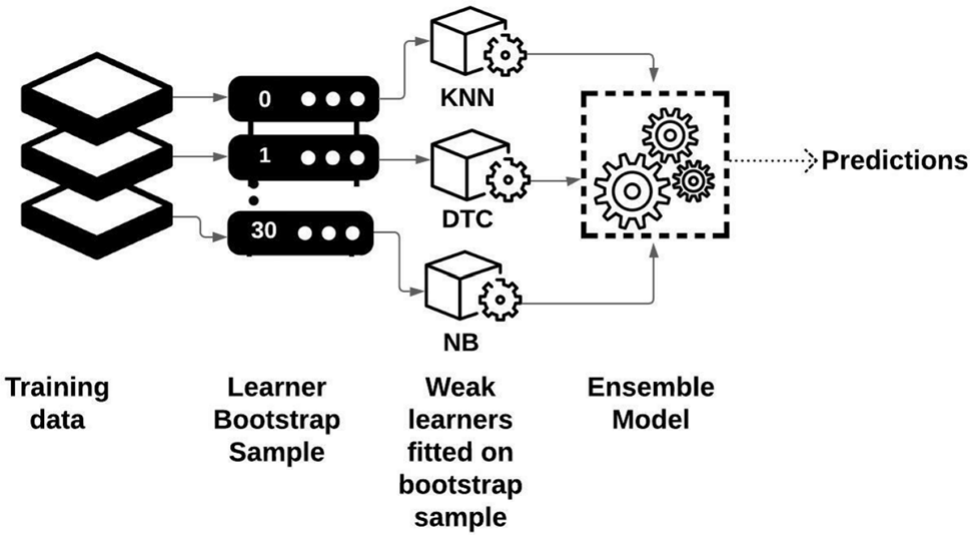
30 Learners used in ensembler.

Ensemble Model that we used for our problem, we constructed a Bagged Tree Ensembler as shown in Fig. 5, using decision tree learner by implementing two steps for solving our problem. Initially, number of base learners are produced using a parallel style manner.

We used Bagging (Bootstrap Aggregation) to reduce the variance of a decision tree. Several subsets of dataset from randomly chosen training samples were created. Then each subset of data was used to train its own decision tree. The weak learners were fitted on the bootstrap. At the end we got a final ensemble comprised of different models. All different trees were averaged with different statistical properties in order to effectively reduce variance and achieve more robustness than a single decision tree.

The idea was to design a base learner that would be accurate and diverse in order to develop a good ensemble^34^.

## Experimental setup and results

In this section we have described the performance of algorithms. We executed the experiments using ML toolbox in MATLAB R2020a. First step was to collect the real data from an authentic source that we managed to do with the collaboration of AZCOM Technology, Italy. AZCOM provided us with the sample dataset comprised of thousands of data values. The collected data was then divided into training and testing dataset in 7:3 ratio i.e. 70% of the total samples were used to train the models and 30% were kept aside for testing purpose. Later the tested values used to check the accuracy level for the designed model, showing accurate predictions. Initially, we selected five features of a signal to design our dataset for detecting PRACH preamble, those were missed detection probability, SNR value, altitude of the signal, variance and the threshold value. The SNR was set with two values 10 as low SNR and 20 as high SNR, overall in the system for the generation of signals. Other parameters were collected accordingly from the generated signals. After training and testing the models with the above selected features, results were not satisfactory. The experimental setup was then rearranged by implementing feature selection option in matlab, to reduce number of irrelevant predictors. New dataset was designed with four features namely altitude, variance and threshold with the same SNR values can be seen in Table 3. Repeating the training and testing steps for the models with collected data by scaling the dataset to same scale as shown in Table 4 we managed to obtain higher accuracy.

**Table 3:** Dataset before pre-processing.

Amplitude	Threshold	Variance	SNR	Results
3298626	329832	6.28E$+$17	20	Peak
2498616	249889	6.28E$+$17	20	Peak
2898670	289836	4.16E$+$17	10	False
2698600	260566	4.93E$+$17	20	False
2814292	281403	5.32E$+$17	10	Peak
.	.	.	.	.
.	.	.	.	.
.	.	.	.	.
2798670	279836	5.16E$+$17	10	Peak
2398600	230566	3.93E$+$17	10	False
2814292	281403	5.32E$+$17	20	Peak

**Table 4:** Final dataset after pre-processing and features scaling.

Amplitude	Threshold	Variance	SNR	Results
$-$ 0.3836	$-$ 0.2618	$-$ 0.9923	$-$ 0.4022	Peak
$-$ 0.3846	$-$ 0.4038	1.9923	$-$ 0.4022	Peak
$-$ 0.3844	$-$ 0.4038	$-$ 0.3085	$-$ 0.3899	False
$-$ 0.3840	$-$ 0.4025	1.9925	$-$ 0.4022	False
$-$ 0.3845	$-$ 0.2055	1.9925	$-$ 0.3899	Peak
.	.	.	.	.
.	.	.	.	.
.	.	.	.	.
$-$ 0.3839	$-$ 0.4038	2.0923	$-$ 0.3899	Peak
$-$ 0.3844	$-$ 0.4025	1.9925	$-$ 0.3899	False
$-$ 0.3836	$-$ 0.2055	$-$ 0.3085	$-$ 0.4022	Peak

**Table 5:** Comparison of proposed methodology with benchmark work.

	Problem	Algorithm	Methodology	Results	Defects
Naresh^11^	Preamble detection in PRACH signal for 5G using supervised machine learning algorithms	Naive bayes K-nearest neighbors	Euclidean distance K = 4	99% detection probability at SNR = − 14.2 db	Dataset was not preprocessed dataset was small CSV data format discrete values
Proposed methodology	Preamble detection in PRACH signal for %G using supervised & Unsupervised machine learning algorithm	Decision tree classification Naive Bayes K-nearest neighbors Ensemble methods	Euclidean distance K=10 DTC learners= 30	97% detection probability at SNR values ranging from 10 to 20	

DTC:

- Maximum Number Of Splits = 4
- Split Criterion = Gini’s diversity index
- Preset = Coarse

**Figure 6:**
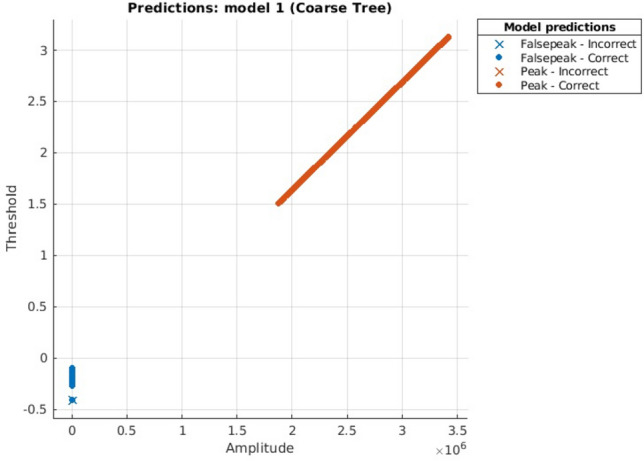
DTC scatter plot.

NB:

- Distribution = Kernel Naive Bayes
- Kernel Type = Gaussian
- Preset = Kernel & MVMN

**Figure 7:**
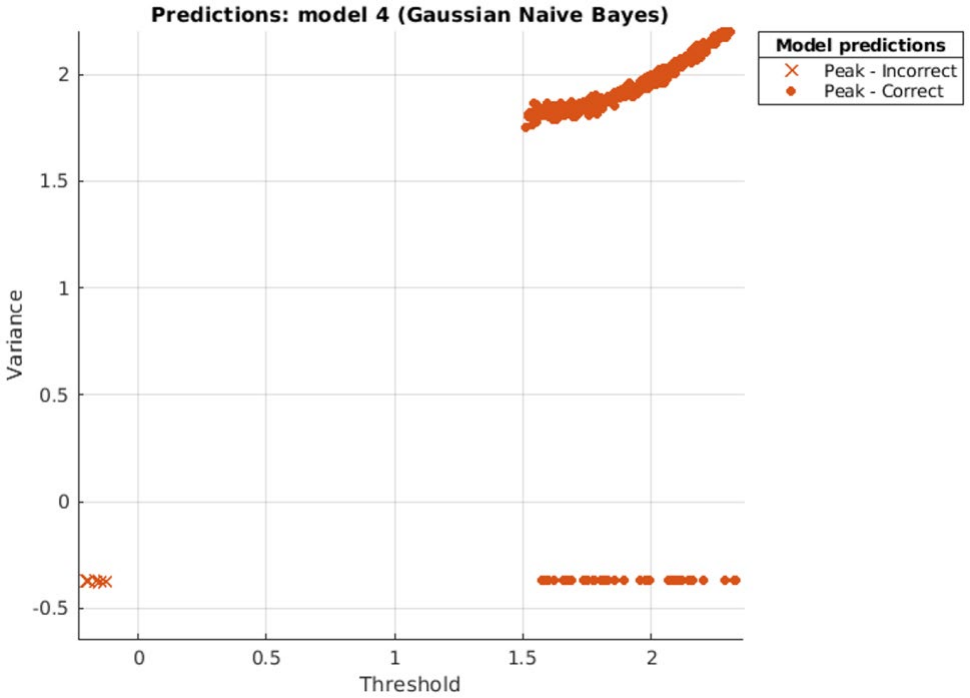
NB scatter plot.

KNN:

- Number Of Neighbors = 10
- Distance Metric = Euclidean
- Preset = Medium

**Figure 8:**
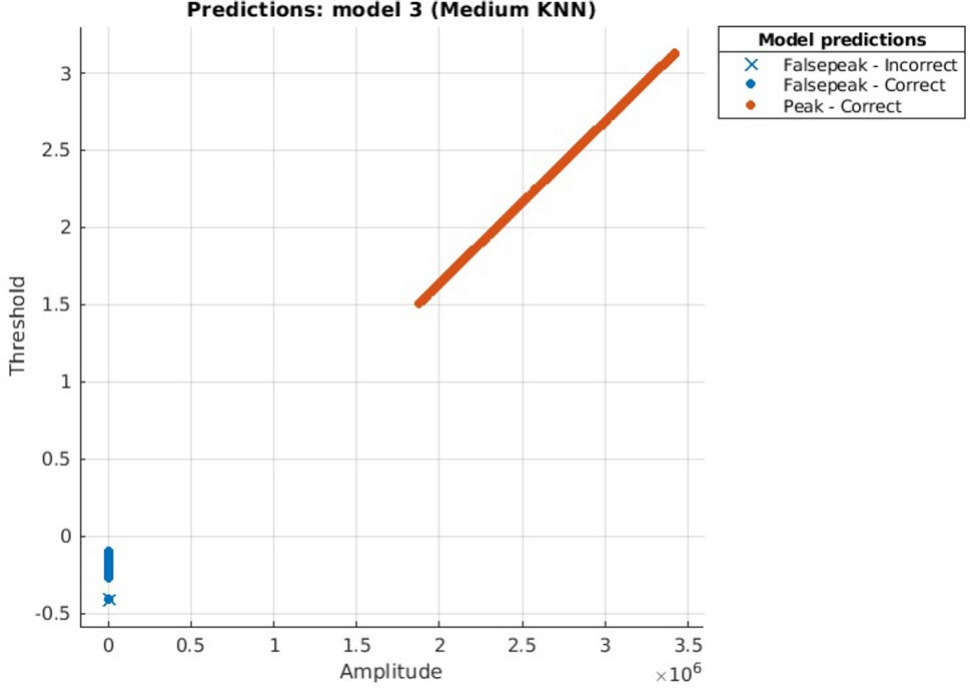
KNN scatter plot.

ENSEMBLE model:

- Preset = Bagged Tress
- Ensemble method = Bag
- Learner type = Decision tree
- Number of learners = 30

**Figure 9:**
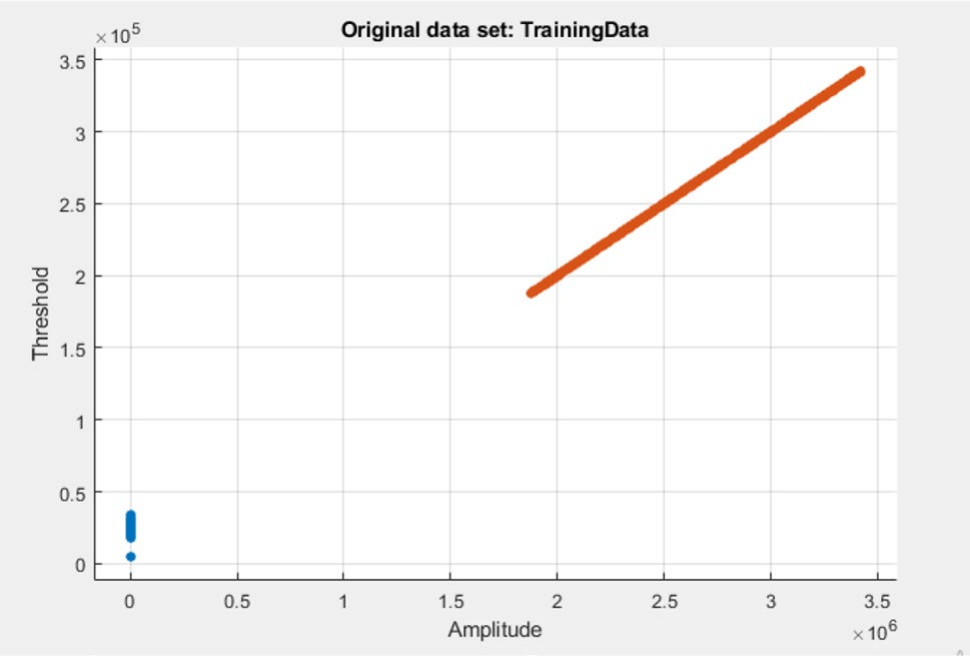
ENSEMBLE scatter plot.

Scatter Plots are showing the separate classes in the graph, where in all graphs it can be seen clearly that both the classes are too far away that these can be easily classified and resulting in highly accurate prediction.

Whereas Parallel Coordinates Plot is used for feature selection, it shows the dependency and effect of one feature over the other, helps in selecting the best fit features for the model.

In the implementation of these techniques we have considered Amplitude, variance of the power delay profile in the current detection window, SNR value and the Threshold as input variables and preamble or false peak as the response in accordance. DTC has a mechanism very similar to binary classification where all the resulting classes are used to arrange the whole decision tree by calculating information gain and entropy at each node. DTC is fast and good for the scenarios where there are less number of predictors available. Comparatively Naive Bayes is also an SL technique similar to DTC, used on discrete values, has low computational cost, efficiently works on large datasets, the only difference here is that the features are treated as independent and probability calculation is done on each feature independently. We have selected Gaussian distribution method to calculate probabilities of features. Furthermore we have used the third algorithm named as KNN, where k is the number of neighbors selected to predict outcome of a particular value. Number of k that we have chosen is 10, predictions were made considering ten nearest values and for distance measurement we used Euclidean method. For Ensemble Method we used 30 DTC learners here as trainer and for predicting more accurate and robust results we implemented bagged tree method.

### Comparison with the benchmark work

To compare our results, we selected^11^ as a benchmark work. The work done by the author is a machine learning based design of PRACH receiver in 5G. The data set used by in this work was not preprocessed. The data set was small hence ML could not be effectively applied. the comparison is shown in Table 5. In our work, we have made a more efficient machine learning model for preamble detection in 5G PRACH. We have preprocessed the data set before training it. The data set passed all the stages of feature engineering. We applied Principal Component Analysis (PCA) so that we only used quality and relatable data. The data set used is finely tuned which gives robust results. The data set is large in size with 1 lac 50 thousand data values from real time 5G system. We fed data set in the model to make its learning more efficient. In implementing our model, we used three supervised learning algorithm i.e, Decision Tree Classification, Naïve Bayes and K-Nearest Neighbor and then an ensemble method too for implementing a hybrid approach for making the prediction more accurate.

## Conclusion

The principle purpose of this research is to compare Ensemble methods with other machine learning algorithms in 5g prachpreamble detection. We have evaluated the efficiency of three supervised learning algorithms on 5G PRACH preamble detection. The results showed that , the accuracy of a ML algorithm is based on two factors; either the training speed or the predicting speed. Here, comparatively DTC consumes less time for training the model but takes more time for predicting, whereas NB trains slowly but predicts exceptionally fast as compared to the other models in this scenario, lastly KNN is overall moderate in both training data and predicting result.

The computational or Prediction speed is one of the distinguishing factors here. According to the experiments done above we observed that, for the same set of dataset consisting of a total of 1,500,000 data values, DTC predicted a number of 470,000 observations per second, this result is visualized in Fig. 6 whereas NB predicted 1600, as shown in Fig. 7 and KNN predicted 130,000 observations per second, as envisioned in Fig. 8. During the experiments, it was noted that prediction Speed of Ensemble model is 21,000 observations per second, as seen in Fig. 9. The results generated and depicted through the graphs show that, both the labels are separated, shown by blue and ornage dots. This distinction shows the accuracy of the algorithm. In this respect we observed, as ensemble model has used 30 learners for training, comparatively it is much faster than other methods.

For future work we would prefer to use other ensemble methods and try to redesign the dataset such that reinforcement learning methods could be implemented.
